# In vitro and in vivo downregulation of the ATP binding cassette transporter B1 by the HMG-CoA reductase inhibitor simvastatin

**DOI:** 10.1007/s00210-015-1169-3

**Published:** 2015-08-30

**Authors:** Bihter Atil, Evelyn Berger-Sieczkowski, Johanna Bardy, Martin Werner, Martin Hohenegger

**Affiliations:** Institute of Pharmacology, Center for Physiology and Pharmacology, Medical University of Vienna, Währingerstraße 13A, 1090 Vienna, Austria; Department of Neurology, Medical University of Vienna, Vienna, Austria; Department of Internal Medicine, Hanuschkrankenhaus, Heinrich-Collin-Strasse 30, 1140 Vienna, Austria

**Keywords:** ABC-transporter, Apoptosis, Dolichol, Glycosylation, HMG-CoA reductase inhibitors, Neuroblastoma, Rhabdomyosarcoma

## Abstract

Extrusion of chemotherapeutics by ATP-binding cassette (ABC) transporters like ABCB1 (P-glycoprotein) represents a crucial mechanism of multidrug resistance in cancer therapy. We have previously shown that the 3-hydroxy-3-methylglutaryl coenzyme A (HMG-CoA) reductase inhibitor simvastatin directly inhibits ABCB1, alters the glycosylation of the transporter, and enhances the intracellular accumulation of doxorubicin with subsequent anti-cancer action. Here, we show that simvastatin reduces endogenous dolichol levels and ABCB1 in human neuroblastoma SH-SY5Y cells. Coapplication with dolichol prevents the downregulation of the ABCB1 transporter. Importantly, dolichol also attenuated simvastatin-induced apoptosis, unmasking involvement of unfolded protein response. Direct monitoring of the fluorescent fusion protein YFP-ABCB1 further confirms concentration-dependent reduction of ABCB1 in HEK293 cells by simvastatin. In simvastatin-treated murine xenografts, ABCB1 was also reduced in the liver and rhabdomyosarcoma but did not reach significance in neuroblastoma. Nevertheless, the in vivo anti-cancer effects of simvastatin are corroborated by increased apoptosis in tumor tissues. These findings provide experimental evidence for usage of simvastatin in novel chemotherapeutic regimens and link dolichol depletion to simvastatin-induced anti-cancer activity.

## Introduction

The ATP-binding cassette (ABC) transporters are one of the largest families of transmembrane proteins and dispose xenobiotics, lipids, and metabolic products across the plasma membrane, mainly in an ATP-dependent manner (Dean and Annilo 2005; Fletcher et al. 2010). The overexpression of ABC transporters is generally associated with resistance to chemotherapy, which is prominently mediated by transporters like ABCB1 (P-glycoprotein; MDR1), ABCC1, and ABCG2 (Gottesman et al. 2002; Cascorbi 2006; Robey et al. 2007; Fletcher et al. 2010). ABCB1 is heavily glycosylated at asparagine residues 91, 94, and 99, which are not affecting transport activity (Schinkel et al. 1993; Gribar et al. 2000; Kvackajova-Kisucka et al. 2001; Dean and Annilo 2005; Seres et al. 2011). Several studies have shown that glycosylation is important for protein quality control in the endoplasmic reticulum (ER) and trafficking to the plasma membrane (Schinkel et al. 1993; Loo and Clarke 1998). Noteworthy, inhibition of glycosylation of ABCB1 induced increased sensitivity to different drugs (Hiss et al. 2007).

Three generations of ABCB1 inhibitors were developed to counteract transporter-driven multidrug resistance in tumors (Gottesman et al. 2002; Fletcher et al. 2010). At last, third-generation compounds tariquidar, elacridar, and others are specific and potent inhibitors of ABCB1, but due to severe side effects and limited efficacy, clinical trials were declined (Fox and Bates 2007; Abraham et al. 2009; Cripe et al. 2010). Thus, it has been postulated that downregulation of ABCB1 or inhibition of compensatory upregulation may represent a novel pharmacological access to transporter-mediated multidrug resistance (MDR) (Tamaki et al. 2011; Amiri-Kordestani et al. 2012).

Statins inhibit 3-hydroxy-3-methylglutaryl coenzyme A (HMG-CoA) reductase and thereby block the mevalonate pathway, which results in reduced levels of cholesterol, farnesyl pyrophosphate (FPP), and geranylgeranly pyrophosphate (GGPP) (Goldstein and Brown 1990). Based on this mechanism of action, statins are widely used in the treatment of cardiovascular diseases accompanied by hypercholesterolemia (Demierre et al. 2005; Gazzerro et al. 2012; Taylor et al. 2013). Beside this cholesterol-lowering effects, so-called pleiotropic effects have emerged, which are in part explained by reduction of intermediates of the mevalonate pathway (Goldstein and Brown 1990; Takemoto and Liao 2001; Gazzerro et al. 2012). The isoprenoids FPP and GGPP are involved in the posttranslational modifications of several proteins, such as small G proteins, which play a critical role in maintenance of cell shape, motility, differentiation, and proliferation (Demierre et al. 2005; Gazzerro et al. 2012). Moreover, dolichol, a side product of the mevalonate pathway, is an important lipid carrier for the glycan precursor in *N*-linked glycosylation of proteins in the ER (Behrens and Leloir 1970; Cantagrel and Lefeber 2011).

Statins’ anti-tumor effects have been investigated in many cellular systems (Farmer 2000; Werner et al. 2004; Demierre et al. 2005; Minichsdorfer and Hohenegger 2009; Sieczkowski et al. 2010; Gazzerro et al. 2012; Chang et al. 2013). Previous studies indicate that human melanoma, neuroblastoma, and rhabdomyosarcoma cells are susceptible to statin-induced apoptosis via the mitochondrial pathway (Werner et al. 2004, 2013; Minichsdorfer and Hohenegger 2009; Sieczkowski et al. 2010). Indeed, statins also directly inhibit ABCB1 in many cellular systems, e.g., with an IC_50_ of 9 μM for simvastatin or 26 μM for lovastatin (Wang et al. 2001; Goard et al. 2010; Martirosyan et al. 2010; Sieczkowski et al. 2010; Werner et al. 2013). The coapplication of simvastatin or lovastatin with doxorubicin, a well-known ABCB1 substrate, increased the accumulation of the anthracycline in many cellular systems and resulted in enhanced nuclear accumulation, potentiated DNA damage, and apoptosis (Goard et al. 2010; Martirosyan et al. 2010; Sieczkowski et al. 2010; Werner et al. 2013). Beside this immediate blockade of ABCB1, we also observed reduced levels of ABCB1 in membranes from simvastatin-treated rhabdomyosarcoma and neuroblastoma cells (Sieczkowski et al. 2010; Werner et al. 2013). The downregulation of ABCB1 affected mainly the fully glycosylated 170-kDa band compared to the core-glycosylated 140-kDa band. This is a clear indication for an involvement of statins in the glycosylation of the ABCB1 transporter (Sieczkowski et al. 2010). Finally, downregulation of ABCB1 by a preincubation with simvastatin was sufficient to result in reduced calcein efflux rates in rhabdomyosarcoma (RD) cells (Werner et al. 2013).

Hence, the aim of this study was to show whether statin exposure to human neuroblastoma cells has an impact on endogenous dolichol levels and whether coadministration of dolichol could prevent ABCB1 downregulation and apoptosis. Moreover, induction of apoptosis and reduction of ABCB1 by simvastatin was evaluated in vivo by murine xenograft models with rhabdomyosarcoma and neuroblastoma cells.

## Materials and methods

### Chemicals and reagents

Simvastatin was purchased from Merck (Darmstadt, Germany), and all other reagents and chemicals from Sigma-Aldrich (St. Louis, MO, USA) or Carl Roth (Karlsruhe, Germany), if not otherwise stated.

### Cell culture

Experiments were performed with human neuroblastoma (SH-SY5Y), rhabdomyosarcoma (RD), and human embryonic kidney (HEK)-293 cells (ATCC–LGC Standards, Wesel, Germany). SH-SY5Y cells were maintained in Dulbecco’s modified Eagle’s medium (DMEM)/Ham’s F12 medium, HEK-293, and RD cells in DMEM high glucose medium, all supplemented with 10 % fetal bovine serum and 1 % penicillin/streptomycin and kept in humidified atmosphere of 5 % CO_2_ at 37 °C.

### Lipid extraction and chromatography

SH-SY5Y cells (1 × 10^7^) were treated according to the figure legend and lysed in methanol plus 3 % acetic acid (*v*/*v*), supplemented with the same volume of hexane and vortexed vigorously. Lipid extraction into the upper phase was repeated and collected, and the organic solvent evaporated. The collected lipids were dissolved in CHCl_3_ and separated by thin-layer chromatography (TLC) using TLC Silica gel 60 plates (Merck; Darmstadt, Germany) and hexane including 20 % ethyl acetate as mobile phase. The separated lipids were visualized by CuSO_4_ and heating of the plate. C_80-105_ dolichol, isolated from bovine heart, and C_55_ dolichol were used as standards. Samples corresponding to the relative motility of the dolichol standards were scratched and transferred to a glass column retaining the matrix of the TLC. Dolichol elution from the silica gel was accomplished by 500 μl CHCl_3_ and repeated four times. The eluate was collected, evaporated, dissolved in 20 μl 2-propanol/methanol/n-hexane (45:45:10), and applied to high-performance liquid chromatography (HPLC). Dolichol fractions were separated with the running buffer (propanol/methanol/n-hexane, 45:45:10) at 1.7 ml/min (Hitachi pump L-2130 and UV detector L-2400; Tokyo, Japan) using C_55_ dolichol or cholesterol as an internal standard. Peaks were analyzed with Elite LaChrom software (Hitachi; Tokyo, Japan).

### Cell lysis and membrane extraction

Untreated and simvastatin-exposed SH-SY5Y cells were washed with phosphate-buffered saline (PBS), shock-frozen with liquid nitrogen, and lysed in RIPA buffer (50 mM Tris-HCl, pH 8.0; 150 mM NaCl; 10 mM glycerophosphate; 0.1 % SDS; 1 % NP-40) containing protease inhibitors aprotinin (2 μg/ml), leupeptin (10 μg/ml), and pefablock (1 mM). Phosphatase inhibitors NaF (1 mM) and Na_3_VO_4_ (1 mM) were supplemented to detect phosphorylated protein species. After 10 min on ice, cells were again shock-frozen in liquid nitrogen and centrifuged with 30000×*g* for 30 min at 4 °C. The supernatant fractions were used for Western blots. Protein concentrations were determined with Bradford protein assay using bovine serum albumin as a protein standard (Bradford 1976).

### Caspase 3 activity

The fluorescence-based caspase 3 assay was carried out as previously described (Sacher et al. 2005; Werner et al. 2013).

### PCR

Following drug treatment, total RNA from SH-SY5Y cells was isolated using RNeasy Mini Kit (Qiagen, Hilden, Germany). After reverse transcription (RevertAid First Strand cDNA Synthesis Kit; Thermo Scientific; Waltham, MA, USA), the cDNA was used for quantitative (real-time) PCR using SensiMix SYBR and Fluorescein (GenXpress, Vienna, Austria) and the specific primers given in Table 1. Quantitative PCR was initiated by a step of denaturation at 94 °C for 3 min, followed by 40 cycles of denaturation at 94 °C for 1 min, annealing at 50 °C for 30 s, and DNA synthesis at 72 °C for 30 s. The final melting step included denaturation at 95 °C for 15 s, 60 °C for 15 s, a linear temperature gradient to 95 °C in 20 min, and 95 °C for 15 s. C_T_ values were normalized to the four control genes (*B2M*, *RPLP0*, *RPS14*, and *GAPDH*), and quantification was performed using the comparative C_T_ method. The PCR for ER stress markers (BiP and CHOP) was run under identical conditions except for 30 cycles, an annealing temperature of 58 °C, and normalization to GAPDH.

**Table 1:** PCR primer sequences

ABCB1	Forward	5′-GCGCCTCGAGATGGATCTTGAAGGGGACC-3′
	Reverse	5′-GCGCGGATCCTGGCGCTTTGTTCCAGC-3′
ABCC1	Forward	5′-CTGACAAGCTAGACCATGAATGT-3′
	Reverse	5′-TCACACCAAGCCGGCGTCTTT-3′
ABCC4	Forward	5′-GGATCCAAGAACTGATGAGTTAAT-3′
	Reverse	5′-TCACAGTGCTGTCTCGAAAATAG-3′
ABCC6	Forward	5′-CACTGCGCTCCAGGATCAGC-3′
	Reverse	5′-CAGACCAGGCCTGACTCCTG-3′
ABCG2	Forward	5′-CTCAGATGGGTTTCCAAGCGTTCATTCA-3′
	Reverse	5′-TGAAACACTGCTTGGTCGTCAGGAAGA-3′
BiP	Forward	5′-CGAGGAGGAGGACAAGAAGG-3′
	Reverse	5′-CACCTTGAACGGCAAGAACT-3′
CHOP	Forward	5′-GCACCTCCCAGAGCCCTCACTCTCC-3′
	Reverse	5′-GTCTACTCCAAGCCTTCCCCCTGCG-3′
B2M	Forward	5′-GTGCTCGCGCTACTCTCTC-3′
	Reverse	5′-GTCAACTTCAATGTCGGAT-3′
RPLP0	Forward	5′-GCAATGTTGCCAGTGTCTG-3′
	Reverse	5′-GCCTTGACCTTTTCAGCAA-3′
RPS14	Forward	5′-GGCAGACCGAGATGAATCCTCA-3′
	Reverse	5′-CAGGTCCAGGGGTCTTGGTCC-3′
GAPDH	Forward	5′-CAAGGTCATCCATGACAACTTTG-3′
	Reverse	5′-GTCCACCACCCTGTTGCTGTAG-3′

### FACS analysis

SH-SY5Y cells (5 × 10^5^) were treated with simvastatin as indicated in figures, and surface ABCB1 transporter was mapped with MRK16 antibody (1:100 dilution in PBS, 30 min at room temperature; Kamiya Biomedical Company, Seattle, WA, USA) and visualized with the corresponding Alexa Fluor^®^488-conjugated goat anti-mouse antibody (1:100 in PBS, 30 min at 4 °C; Invitrogen, CA, USA). Alternatively, C219 (Abcam, Cambridge, UK) or p170 (Neomarkers, Fremont, CA, USA) antibodies (1:50 in 10 % FCS with 1 % NaN_3_ in PBS, 2 h at room temperature) were used for ABCB1 staining in fixed cells and gave similar results. In all FACS experiments, unstained cells and/or cells mapped with mouse IgG_2a_ (Becton Dickinson, Heidelberg, Germany) were used as negative controls to correct for background. The data were processed off-line with Flowing software (www.flowingsoftware.com).

Apoptosis was determined with biparametric FACS analysis using FITC-conjugated annexin V (Ebioscience, San Diego, CA, USA) and propidium iodide (PI) as previously described (Minichsdorfer and Hohenegger 2009).

### Rhodamine 123 efflux

Rhodamine 123 efflux was performed as previously described by Donmez Cakil et al. (Donmez Cakil et al. 2014). Briefly, SH-SY5Y cells (1 × 10^6^) were exposed to increasing concentrations of simvastatin for 48 h, washed, and incubated with 0.53 μM rhodamine123 for 30 min at 37 °C. Fluorescence (excitation at 488 nm and emission wavelength at 534 nm) was continuously monitored (5 min) with a FACSCalibur. Surface expression of ABCB1 was controlled by FACS with MRK16 staining, and off-line analyses were done with CellQuest software (Becton Dickinson, Heidelberg, Germany), as previously described (Chiba et al. 1996; Donmez Cakil et al. 2014).

### Protein turnover of YFP-ABCB1 fusion protein

HEK-293 cells (5 × 10^6^) were transfected with 0.4 μg YFP-ABCB1-pcDNA3 plasmid using TurboFect following the manufacturer’s protocol (Thermo Scientific; Waltham, MA, USA). The NH_2_-terminal-tagged YFP-ABCB1 construct was kindly provided by Prof. Peter Chiba (Institute of Medical Chemistry) and Dr. Oliver Kudlacek (Institute of Pharmacology, Medical University of Vienna). After 48 h of recovery, cells were treated for another 48 h in the absence or presence of cycloheximide (10 μg/ml), doxorubicin (0.1 μM), simvastatin (1 or 3 μM), or vehicle (empty pcDNA3 plasmid). Thereafter, cells were shock-frozen and resuspended in PBS, and the YFP fluorescence (exCitation 515 nm, emission 530 nm) was measured with a fluorescence spectrophotometer (FL-4500 Hitachi; Tokyo, Japan). Signals were corrected for protein concentration and background signal of the vehicle (FL-Solutions 2.0 software; Hitachi; Tokyo, Japan).

### Murine xenograft experiments

Two xenograft experiments were performed and approved by the Animal Welfare Committee of the Medical University of Vienna and the Austrian Science Ministry (GZ66.009/0271-BrGT/2005 and GZ66.009/0274-II/3b/2010). Female CD-1 Nu/Nu mice (6 weeks old; Charles River; Sulzfeld, Germany) were subcutaneously inoculated with SH-SY5Y neuroblastoma cells (1 × 10^7^ in PBS) into the left and right flank. Twelve days after inoculation, groups of four mice were assigned to control group or simvastatin (4.25 mg/kg/day; oral) group. In both groups, one inoculum did not turn into a tumor. A day–night rhythm was emulated by light every 12 h, and the welfare of the animals was checked every day. The animals were killed by neck dislocation after 2 months or earlier due to critical tumor size. Organs and tumors were excised and weighted, and aliquots were rapidly frozen in liquid nitrogen and stored at −80 °C for further analyses.

Under similar conditions, female CD-1 Nu/Nu mice were inoculated with rhabdomyosarcoma (RD) cells (1.5 × 10^6^ cells in PBS) into the right flank. One week after inoculation, animals received water (control; *n* = 10), simvastatin (1.15 mg/kg/day; *n* = 6), cyclophosphamide (2 mg/kg/day; *n* = 6), or a combination of simvastatin and cyclophosphamide (*n* = 6). The animals were killed after 54 days or earlier due to critical tumor size. Tumors and livers were excised, weighted, and fixed in 4 % paraformaldehyde for staining and immunohistochemistry. Small aliquots of organs and tumors were also rapidly frozen in liquid nitrogen and stored at −80 °C for further analyses.

For tissue analysis, liver and tumor samples (25–50 mg) were homogenized in solution A (10 mM HEPES, pH 7.5; 10 % sucrose; 5 mM EDTA; 1 mM DTT; 1 mM pefablock; 100 μM aprotinin; 100 μM leupeptin; 10 μM calpain inhibitors I and II). Lysates were centrifuged at 100×*g* for 5 min, and the supernatant was centrifuged again with 600×*g* for 10 min. The supernatant was again centrifuged at 11,600×*g* for 20 min. At last, the supernatant was centrifuged again at 100,000×*g* for 45 min. Pellet corresponding to the membrane fraction was resuspended in 100–200 μl solution B (10 mM HEPES, pH 7.5; 10 % sucrose; 2 mM EDTA; 1 mM DTT) supplemented with the above protease inhibitors and stored at −80 °C. All steps were carried out at 4 °C.

### Immunohistochemical analysis of tumors

Tumors were embedded in optimal cutting temperature compound (OCT Tissue-Tek, Sanova, Vienna, Austria) prior to frozen sectioning on a microtome cryostat (Microm HM-500-OM, Walldorf, Germany). Alternatively, samples were fixed with 5 % formalin buffered in PBS. Sections were cut (3–5 μm) and formalin was removed by increasing concentrations of ethanol. If necessary, sections were dehydrated by methanol and again hydrated in water. Fixed slices were incubated with 4 % paraformaldehyde and then blocked with 4 % bovine serum albumin in PBS. Nuclei were stained with Hoechst 33258 (15 min) and activated caspase 3 with an antibody selective for cleaved caspase 3 (1:200, overnight; Cell Signaling, Millipore, Vienna, Austria). Immunoreactive species were detected with a corresponding Cy3-conjugated antibody (1:200; excitation 543 nm, emission 570 nm; PA43004, GE Healthcare, Vienna, Austria), and images were taken with a Zeiss fluorescence microscope (Axioimager Z1, Jena, Germany).

### Western blot analysis

Proteins (15–30 μg) were separated on a 7 or 10 % SDS-PAGE, transferred to nitrocellulose membranes, blocked with 5 % bovine serum albumin, and incubated overnight at 4 °C with the following primary antibodies: ABCB1 (C219, 1:300; Merck; Darmstadt, Germany), cleaved poly-ADP ribose polymerase (cleaved PARP, 1:1000; Cell Signaling Millipore, Vienna, Austria), extracellular-signal-regulated protein kinases 1 and 2 (ERK1/2) and phospho-ERK1/2 (1:2000; Cell Signaling Millipore, Vienna, Austria), actin (AC-40, 1:20,000), or α-tubulin (anti-α tubulin, 1:40,000). Proteins of interest were visualized by enhanced chemiluminescence (ECL) system (ECL Plus, GE Healthcare, Vienna, Austria) using a species-corresponding horseradish peroxidase (HRP)-conjugated secondary antibody (1:10,000 in 2 % BSA; Cell Signaling Cell Signaling Millipore, Vienna, Austria) for 1 h at room temperature. Actin and α-tubulin were alternatively used as loading controls depending on compatibility with species of the antibody and molecular mass range of the protein of interest. Three independent experiments were carried out for Western blots, and samples were used up to three times to repeat and optimize X-ray exposure times for ECL detection. Bands of interest were quantified and analyzed using ImageJ software (http://rsbweb.nig.gov/ij/).

### Statistical analysis

Typically, three independent experiments were carried out in duplicates (*n* = 3) and the data are presented as mean ± standard deviation (SD) if not otherwise stated. Statistical analyses were performed using SigmaPlot software (Jandl, Erkrath, Germany) with either unpaired Student’s *t* test or for multiple comparisons ANOVA and Holm–Sidak test or post hoc Tukey test. A *p* value <0.05 is considered statistically significant.

## Results

### ABCB1 downregulation in simvastatin-treated SH-SY5Y cells

**Fig. 1:**
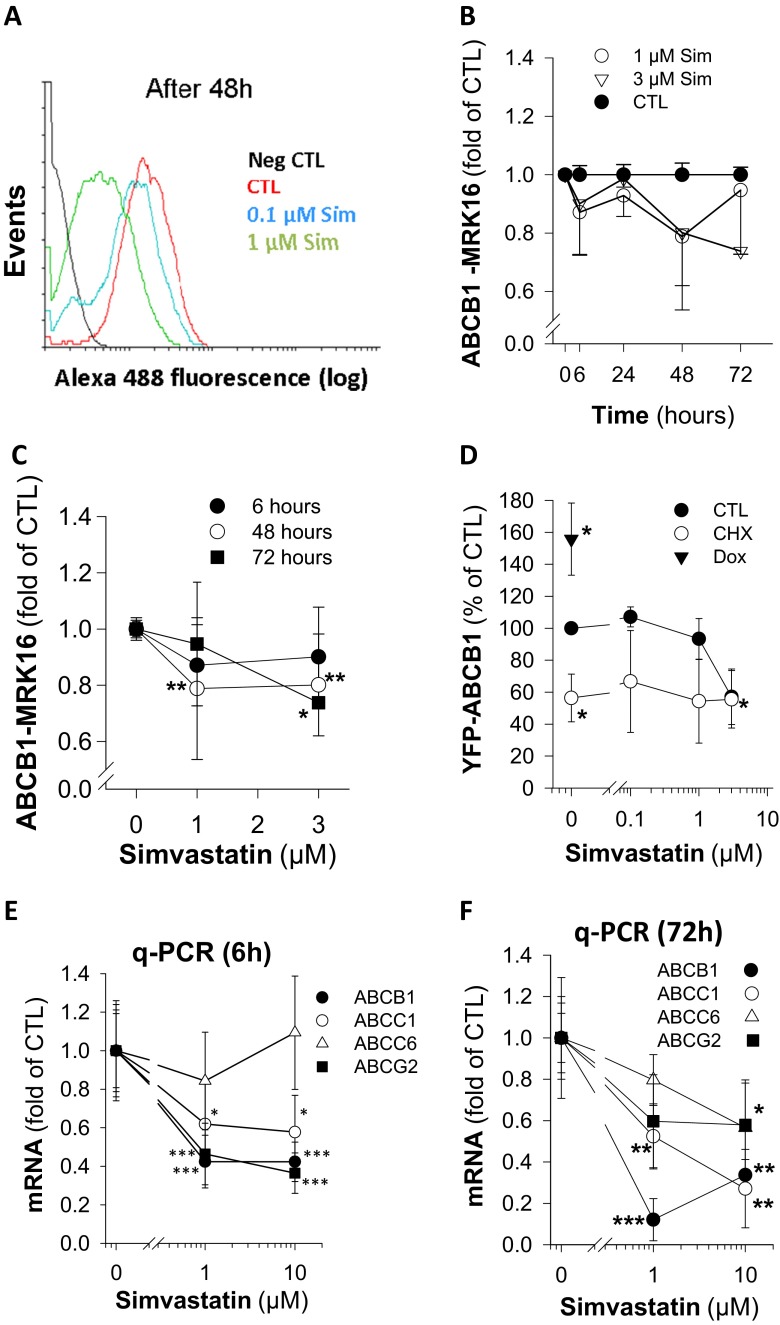
Downregulation of ABCB1 by simvastatin. SH-SY5Y cells were exposed to the indicated simvastatin (Sim) concentrations and compared to controls (CTL). Cell surface expression of ABCB1 was monitored by FACS analysis with the MRK16 antibody (**a**–**c**). A histogram is depicted in panel (**a**), the kinetics are given in panel (**b**) (*n* = 3) and concentration dependency in panel (**c**) (*n* = 3). HEK-293 cells were transfected with YFP-ABCB1-pcDNA3, and after 48 h, the cells were exposed to doxorubicin (Dox; 0.1 μM), cycloheximide (CHX; 10 μg/ml), and various concentrations of simvastatin for additional 2 days (**d**) (*n* = 3). Quantitative PCR of various ABC transporters is depicted from simvastatin-treated SH-SY5Y cells for 6 h (**e**) or 72 h (**f**) (*n* = 6–12). All values were presented as mean ± SD and corrected for background fluorescence. Significance was tested with one-way ANOVA (Holm–Sidak method). *Asterisks* denote significance versus control (*p < 0.05; **p < 0.005; ***p < 0.0005)

Simvastatin reduces the fully glycosylated form of the ABCB1 transporter in rhabdomyosarcoma (RD) and SH-SY5Y neuroblastoma cells shown by Western blots (Sieczkowski et al. 2010; Werner et al. 2013). In order to quantify this effect, we here confirm significant reduction of cell surface ABCB1 protein expression in simvastatin-treated SH-SY5Y neuroblastoma cells by FACS analysis (Fig. 1a–c). The kinetics of cell surface reduction of ABCB1 is detected with MRK16 antibody in non-permeabilized SH-SY5Y neuroblastoma cells. ABCB1 reduction is hardly observed after 24-h simvastatin exposure (Fig. 1b), indicating an early compensatory mechanism. However, a significant reduction of ABCB1 surface expression is observed after 48 h with already 1 μM simvastatin (Fig. 1c).

In order to further confirm ABCB1 downregulation by simvastatin in another experimental setting including also intracellular ABCB1, the fluorescent fusion protein YFP-ABCB1 was heterologously expressed in HEK-293 cells (Fig. 1d). This experimental setup was validated by doxorubicin as a positive control for upregulation of the ABC transporter and cycloheximide as a negative control mirroring transcriptional inhibition. A concentration of 3 μM simvastatin was sufficient to reduce the transporter within 48 h significantly. Simvastatin had no effect on the fluorescence signal of YFP-ABCB1 in the presence of cycloheximide, a blocker of translational elongation. Accordingly, inhibition of transcription is considered to be responsible for the simvastatin-dependent reduction of ABCB1 protein. These findings together with previous reports confirm that simvastatin in the low micromolar concentration range downregulates ABCB1 on protein level, which accounts for a 20–40 % reduction, depending on the experimental conditions (Fig. 1) (Sieczkowski et al. 2010; Werner et al. 2013).

Accordingly, one would expect a decrease of ABCB1 messenger RNA (mRNA) levels in the presence of simvastatin. The tissue distribution, spectrum of substrates and regulators, and the role in multidrug resistance are overlapping for ABCC1 and ABCG2 with ABCB1 (Sharom 2008). In contrast, little is known about ABCC6 in regard to multidrug resistance and compensation of ABCB1 downregulation (Vanakker et al. 2013). A significant reduction of ABCB1, ABCC1, and ABCG2 mRNA is already seen after 6 h of simvastatin exposure, which is further enhanced upon longer incubation times (Fig. 1e, f). The amount of ABCC6 mRNA is not affected and documents specificity of the simvastatin effect. Most prominently, mRNA of ABCB1 and ABCC1 is already downregulated by 1 μM simvastatin. Noteworthy, these findings are not indicative for a compensatory upregulation of alternative transporters like ABCC1 and ABCG2.

### Transporter activity of ABCB1 is affected by simvastatin

**Fig. 2:**
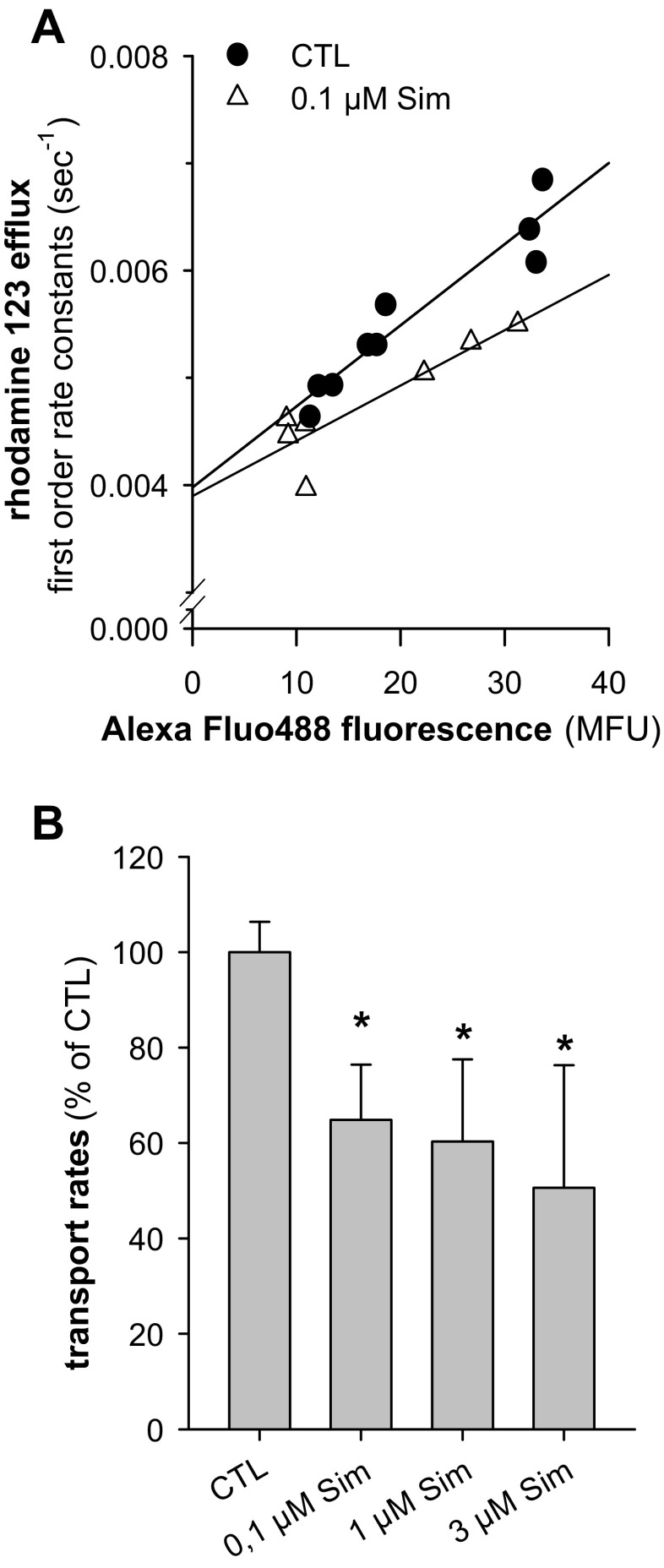
Simvastatin reduces rhodamine 123 transport rates in SH-SY5Y cells. SH-SY5Y cells were treated with the indicated simvastatin (Sim) concentrations for 48 h and loaded with rhodamine 123. The rhodamine 123 efflux was monitored by FACS analysis and fitted to a mono-exponential decay, and the corresponding first-order rate constants were plotted against the fluorescence of the MRK16/Alexa Fluo488 fluorescence, which was used as a measure for ABCB1 surface expression of the corresponding measurement (mean fluorescence units, MFU). The data points represent nine individual measurements of four control experiments (CTL) and seven individual measurments of three experiments in the presence of 0.1 μM simvastatin (Sim) (**a**). The transportation rates in the absence (CTL) and presence of simvastatin (Sim) (**b**). Data represent mean ± SD (*n* = 4; 0.1 μM Sim, *n* = 3; *p < 0.05)

Functionally, statins directly inhibit ABCB1 (Bogman et al. 2001; Wang et al. 2001; Goard et al. 2010; Martirosyan et al. 2010; Sieczkowski et al. 2010; Werner et al. 2013). In order to confirm previous findings using calcein-AM, we here determined first-order rate constants of rhodamine 123 efflux which were plotted against the corresponding expression of ABCB1. This approach allowed correcting for different expression levels of the transporter, which was reduced in the presence of simvastatin (Fig. 2a). The slopes of such linear regressions yield rhodamine 123 transportation rates (Fig. 2b), which were significantly reduced by simvastatin in a concentration-dependent manner. However, a significant difference between simvastatin concentrations was not obtained.

### Simvastatin depletes cells from endogenous dolichol

**Fig. 3:**
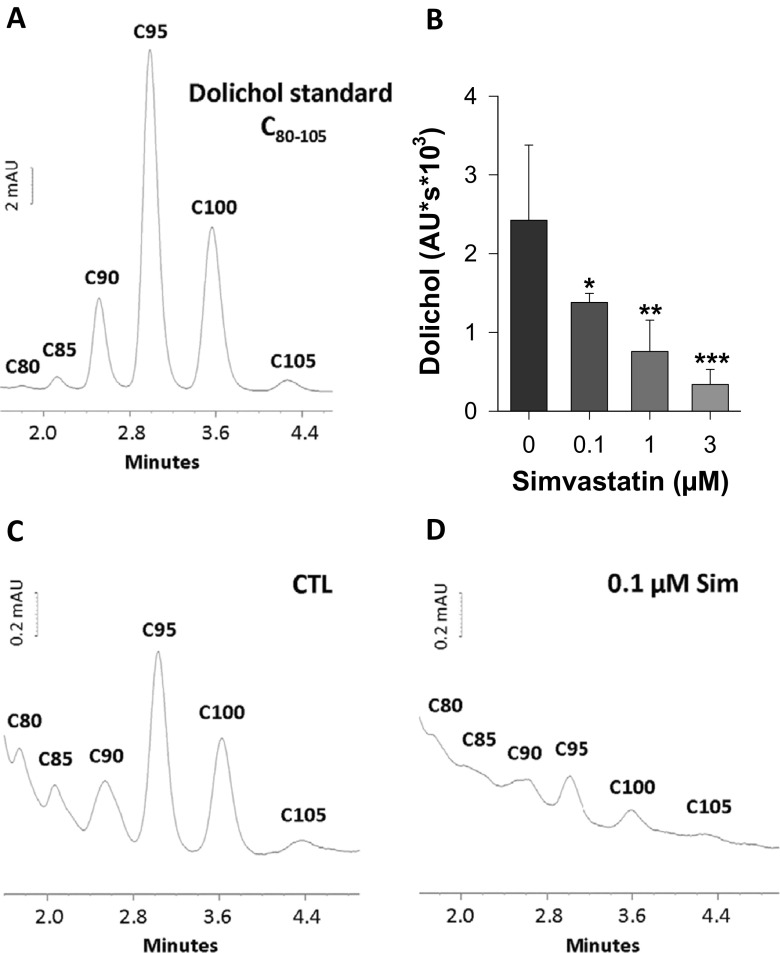
Simvastatin depletes SH-SY5Y cells from endogenous dolichol. HPLC analysis of dolichol C_80-105_ standard (**a**) was compared with endogenous dolichol levels in SH-SY5Y cells of controls (CTL) (**c**) and simvastatin-treated cells (48 h) (**d**). Absorption units denote AU and mAU. The areas under the curve of the dolichol C_80-105_ peaks were used to quantify the simvastatin-induced effects (**b**) (*n* = 5; *p < 0.05, **p < 0.005, ***p < 0.0005)

In support to our conjecture, that glycosylation of proteins is impaired by simvastatin exposure, we have determined endogenous dolichol levels in SH-SY5Y cells (Fig. 3) (Sieczkowski et al. 2010). Expectedly, simvastatin exposure reduced endogenous dolichol levels in a concentration-dependent manner (Fig. 3b). Noteworthy, a significant reduction of dolichol was observed already at 0.1 μM simvastatin compared to untreated cells (Fig. 3c, d).

**Fig. 4:**
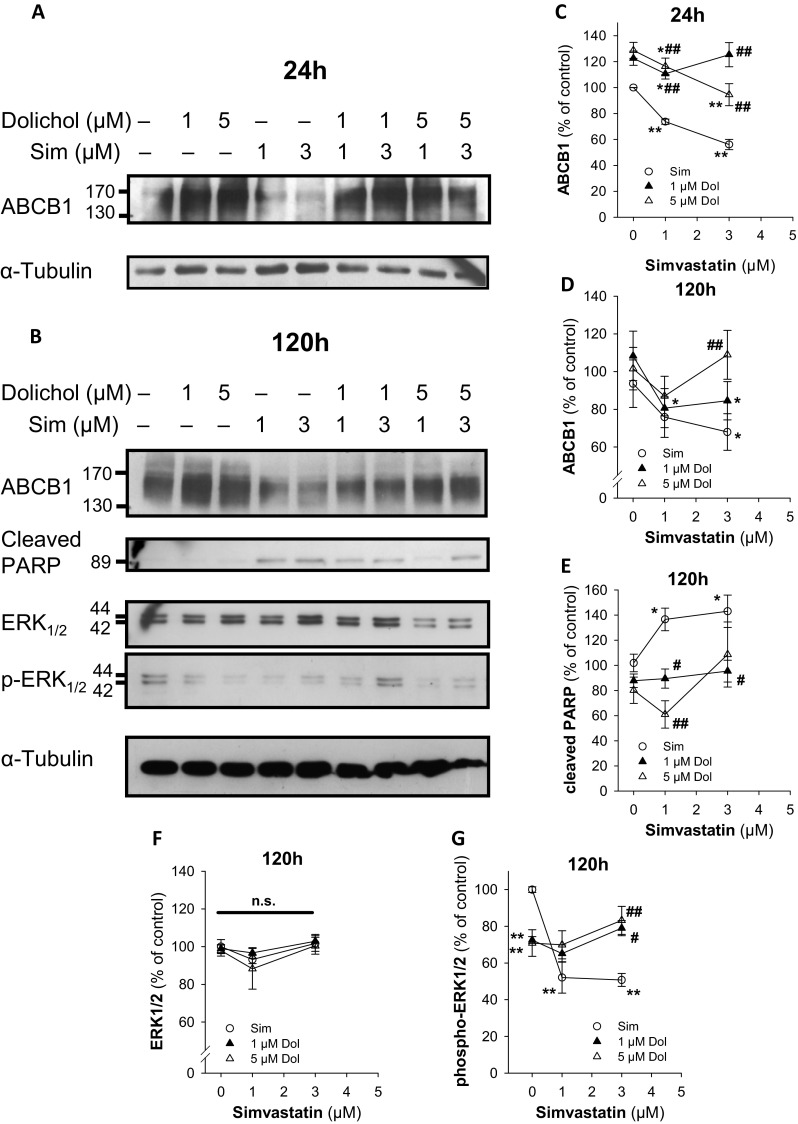
Dolichol restores simvastatin-induced ABCB1 downregulation and prevents apoptosis. SH-SY5Y cells were treated with simvastatin (Sim) in the absence and presence of dolichol C_80-105_ (Dol) for 24 (**a**, **c**) or 120 h (**b**, **d**–**g**) and probed for ABCB1, cleaved PARP, ERK1/2, and phosphorylated ERK1/2. α-Tubulin was used as a loading control and the molecular mass of the protein standards or proteins of interest are indicated (kDa). Western blots show a representative experiment, which was repeated two times. Panels (**c**)–(**g**) depict the densitometric analyses of the proteins of interest as shown in a representative experiment in panels (**a**) and (**b**) (*n* = 3). Intensities were normalized to the loading controls and given as percent of control. *Asterisks* denote significance versus control in the absence of the simvastatin (*p < 0.05; **p < 0.005; n.s. denotes not significant). Hatches denote significance versus the respective simvastatin concentration (^#^ *p* < 0.05; ^##^ *p* < 0.005)

Dolichol phosphate is a downstream product of the HMG-CoA reductase and critically required for glycoprotein biosynthesis (Behrens and Leloir 1970; Goldstein and Brown 1990). One would now postulate that dolichol C_80-105_ coapplication prevents downregulation of ABCB1 by simvastatin (Fig. 4a–d). Interestingly, the addition of dolichol alone, but also in the presence of simvastatin, strongly intensified the ABCB1 bands, indicating full saturation of the glycosylation machinery. Conversely, with simvastatin alone, a significant downregulation of ABCB1 is detectable at 24 h and longer incubation times (120 h, Fig. 4c, d). Importantly, the addition of dolichol significantly protected from simvastatin-induced downregulation of ABCB1 (Fig. 4c, d). The protein bands for ABCB1 are fuzzy and broad, as this is assumed to be due to different levels of glycosylation, in particular in the presence of exogenous dolichol. Similar findings have been described by others previously (Loo and Clarke 1999; Gribar et al. 2000; Zhang et al. 2004; Seres et al. 2011). However, the reduction of ABCB1 is specific since protein levels of α-tubulin and ERK1/2 were not affected by simvastatin or dolichol (Fig. 4f). Moreover, the well-described inhibitory effect of statins on ERK1/2 phosphorylation is seen in simvastatin-exposed probes and significantly reversed by coadministration with dolichol (Fig. 4g) (Campbell et al. 2006). Noteworthy, dolichol per se significantly reduced ERK1/2 phosphorylation, which is currently not understood.

**Fig. 5:**
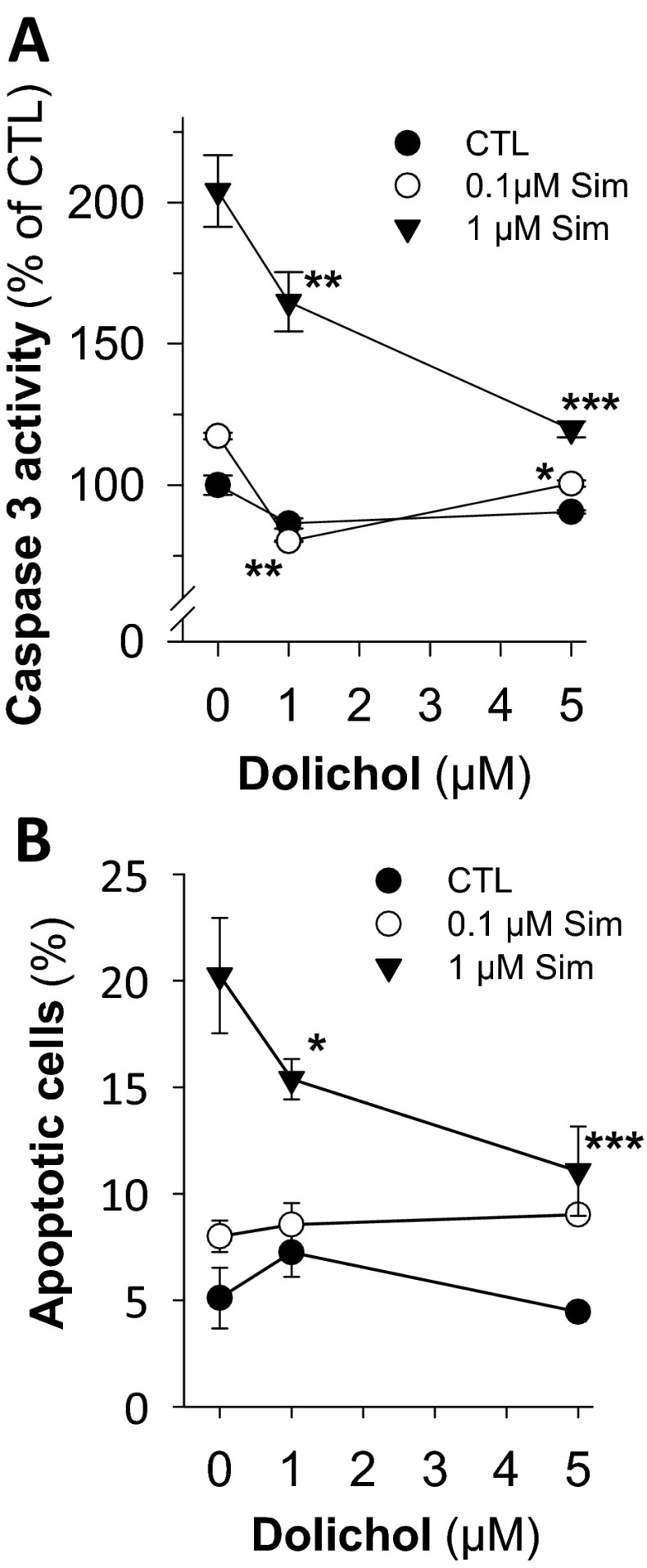
Dolichol restores simvastatin-induced apoptosis. Caspase 3 activation is monitored in lysates from SH-SY5Y cells treated with simvastatin (Sim) in the absence (CTL) and presence of dolichol C_80-105_ for 48 h (**a**). Similar treated cells were analyzed for apoptosis by FACS analysis of annexin V/PI-positive cells (**b**). The *symbols* represent mean ± SD (*n* = 3). *Asterisks* denote significance versus control (*p < 0.05; **p < 0.01; ***p < 0.005)

The dolichol dependency of simvastatin effects was also observed on the level of apoptosis. The cleaved PARP fragment significantly accumulates in simvastatin exposed cell extracts and is reduced by coadministration of dolichol (Fig. 4b, e). This observation is supported by simvastatin-dependent activation of caspase 3 and positivity for annexin V/PI staining, which were again significantly reduced by coadministration with dolichol (Fig. 5). Thus, impairment of glycosylation in the endoplasmic reticulum (ER) leads to reduction of heavily glycosylated proteins but also to cellular stress resulting in apoptosis. Mechanistically, this can be explained by simvastatin-induced ER stress, which is detected by the upregulation of the specific genes like BiP/GRP78 and the transcription factor CCAAT/enhancer-binding protein (C/EBP)-homologous protein (CHOP) (Table 2) (Haataja et al. 2008; van Schadewijk et al. 2012). Already at 0.1 μM simvastatin, BiP and CHOP were significantly induced, while at 10 μM simvastatin, no induction of the ER chaperone BiP was observable. An explanation for this latter observation is currently not available.

**Table 2:** Simvastatin-induced unfolded protein response

Simvastatin (μM)	BiP (a.u.)	CHOP (a.u.)
0	1	1
0.1	1.37 ± 0.10 (**)	1.64 ± 0.12 (**)
1	1.28 ± 0.04 (**)	1.39 ± 0.13 (*)
10	1.06 ± 0.05 (n.s.)	2.35 ± 0.27 (***)

Markers for ER stress, BiP and CHOP, were detected on mRNA level by PCR and normalized to GAPDH values from probes treated with increasing simvastatin concentrations for 48 h (mean ± SD, *n* = 3)

(*p < 0.02; **p < 0.005; ***p < 0.001; n.s. denotes not significant; statistical significance is calculated with one-way ANOVA and Holm–Sidak method

### In vivo effects of simvastatin on tumor growth and ABCB1 expression

In order to confirm in vivo relevance of our findings, CD-1 Nu/Nu mice were inoculated with either SH-SY5Y neuroblastoma cells or RD rhabdomyosarcoma cells, and simvastatin was applied orally, 4.25 or 1.15 mg/kg/day, respectively. These doses of simvastatin translate into a human equivalent dose of 7 and 20 mg, which is a typical starting dose for simvastatin (Reagan-Shaw et al. 2008; Gazzerro et al. 2012).

**Fig. 6:**
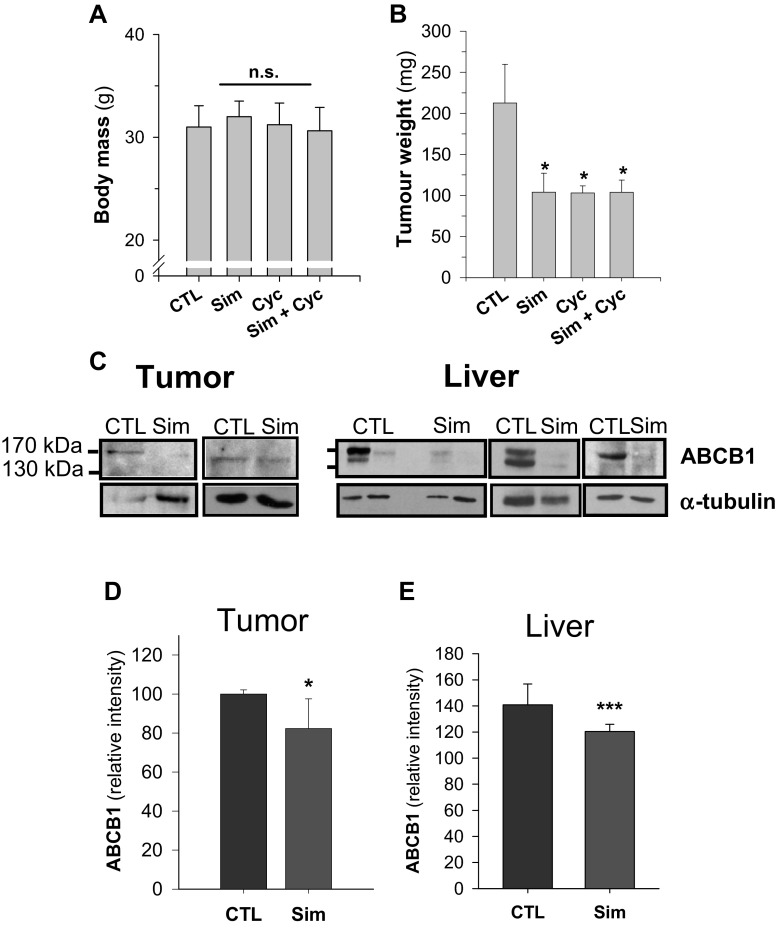
Rhabdomyosarcoma xenograft model. Female CD-1 Nu/Nu mice were inoculated with human RD rhabdomyosarcoma cells and then assigned to the following groups: control (CTL; *n* = 10), 1.15 mg/kg/day simvastatin (Sim; *n* = 6), 2 mg/kg/day cyclophosphamide (Cyc; *n* = 6), and a combination therapy (Cyc + Sim; *n* = 6). The postmortem body mass (**a**) and tumor weight is given as mean ± SD (**b**). Representative Western blot analyses of individual samples of different livers and rhabdomyosarcoma tissues were probed for ABCB1 and α-tubulin as a loading control (**c**). Intensities of protein bands of interest were normalized to the loading control (**d**, **e**). Significance was calculated versus control with one-way ANOVA (Holm–Sidak method) or Student’s *t* test (*n.s*. denotes not significant; *p < 0.05; ***p < 0005)

There was no significant difference between the body mass of control and simvastatin-treated mice in both models (Table 3 and Fig. 6a). In the neuroblastoma xenograft model, there was also no significant difference in organ weights (Table 3), indicating no gross metastatic activity. Conversely, tumor weight in simvastatin-treated mice was diminished in comparison to control animals; however, this decrease was only significant in the RD xenograft model (Table 3 and Fig. 6b).

**Table 3:** Analysis of the neuroblastoma xenograft experiment

	Control	Simvastatin
Liver (*n* = 4)	1.49 ± 0.14	1.59 ± 0.24 (n.s.)
Heart (*n* = 4)	0.14 ± 0.02	0.13 ± 0.01 (n.s.)
Spleen (*n* = 4)	0.19 ± 0.06	0.18 ± 0.05 (n.s.)
Kidney (*n* = 8)	0.53 ± 0.20	0.52 ± 0.10 (n.s.)
Lung (*n* = 4)	0.21 ± 0.05	0.20 ± 0.02 (n.s.)
Tumor (SH-SY5Y cells; *n* = 7)	2.77 ± 1.87	2.25 ± 1.61 (n.s.)
Body weight (*n* = 4)	34.48 ± 4.54	36.06 ± 5.51 (n.s.)

Postmortem, the organs of four mice in each group were weighted (g) and presented as mean ± SD. In both groups, one tumor was not detectable. Comparison of the two groups with Student’s *t* test revealed no statistical significance (n.s.)

**Fig. 7:**
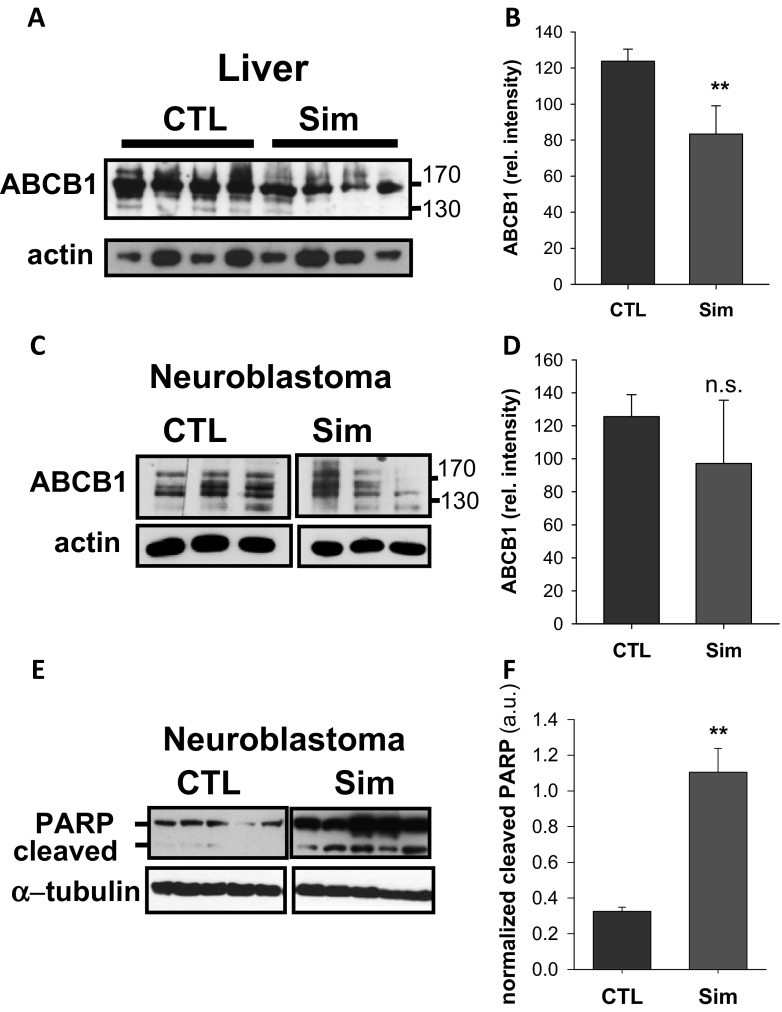
Neuroblastoma xenograft model. Female CD-1 Nu/Nu mice were inoculated with human SH-SY5Y cells and assigned to control (CTL), or 4.25 mg/kg/day simvastatin (Sim) groups of four animals. Postmortem, tissues from liver (**a**, **b**) and neuroblastoma (**c**–**f**) were probed for ABCB1 and PARP, with actin and α-tubulin as a loading control. Representative Western blots are depicted (**a**, **c**, **e**), and the intensities of the proteins of interest were normalized to the loading control of all experiments (*n* = 3–5) (**b**, **d**, **f**). Statistical significance was calculated with Student’s *t* test (n.s. denotes not significant; **p < 0.005)

Following oral administration, simvastatin reaches the highest concentrations in the liver (Gazzerro et al. 2012). Accordingly, ABCB1 was significantly reduced in tissue homogenates from liver of simvastatin-treated animals (Figs. 6 and 7). Moreover, a similar trend was observed in rhabdomyosarcoma and neuroblastoma, which was only significant in rhabdomyosarcoma (cf., Figs. 6 and 7). Taken together, these in vitro findings confirm that simvastatin is able to downregulate ABCB1 also in vivo at pharmacological relevant dosages.

### In vivo detection of simvastatin-induced apoptosis in tumors

**Fig. 8:**
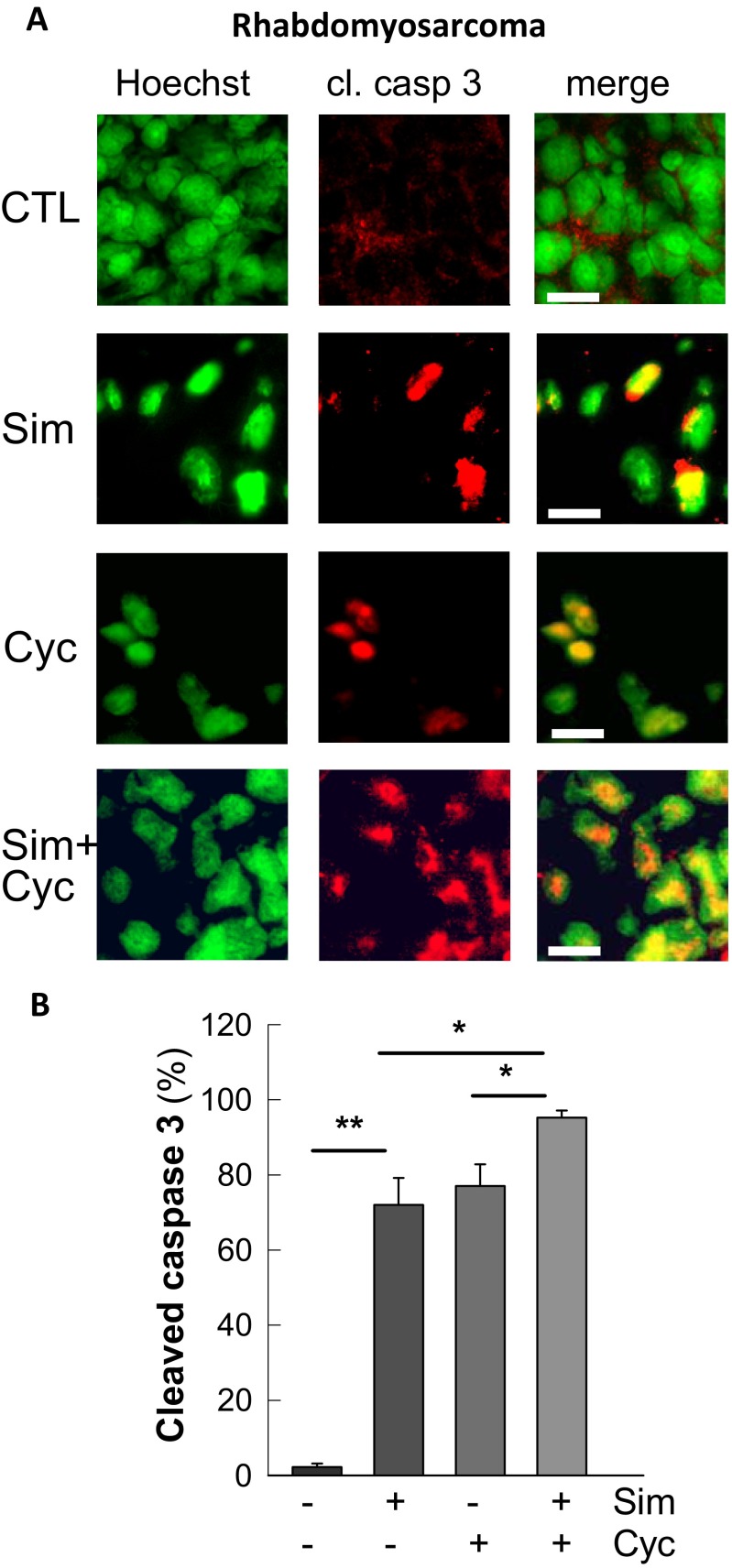
Activation of caspase 3 in rhabdomyosarcoma of simvastatin-treated animals. Rhabdomyosarcomas from animals described in Fig. 5 were used for immunohistochemistry to detect cleaved caspase 3 (**a**) in controls (CTL), simvastatin (Sim)-, and/or cyclophosphamide (Cyc)-treated groups. Nuclei were stained with Hoechst dye (*green*), and cleaved caspase 3 (cl. casp 3; *red*). The merged pictures allow identification of the caspase 3 translocation into the nucleus equivalent to ongoing apoptosis (magnification ×63; *white scale bars*, 20 μm). Nuclei positive for cleaved caspase 3 were counted, and normalized data of 20 pictures from each group are depicted (**b**). Statistical significance for multiple comparisons was calculated with one-way ANOVA and post hoc Turkey test (*p < 0.05; **p < 0.005)

Besides the downregulation of ABCB1 and a direct inhibition of the transporter, simvastatin triggers also apoptosis in rhabdomyosarcoma and neuroblastoma cells (Werner et al. 2004, 2013; Sieczkowski et al. 2010). This pro-apoptotic effect of simvastatin is now also confirmed in vivo. Simvastatin induced significant PARP cleavage in neuroblastoma (Fig. 7e, f) and cleavage of caspase 3 in rhabdomyosarcoma (Fig. 8). In the latter xenograft model, cyclophosphamide was applied as positive control for anti-tumor activity, without being a substrate for ABCB1. Importantly, reduction in tumor mass (Fig. 6b) and the activation of caspase 3 (Fig. 8) were triggered by cyclophosphamide to the same extent compared to simvastatin or even further enhanced by coapplication. Thus, statins like simvastatin might serve as a partner in novel chemotherapeutic combinations even independent of ABCB1 substrates as this has been postulated previously (Demierre et al. 2005; Gazzerro et al. 2012).

## Discussion

Statin treatment alters the glycosylation pattern of ABCB1 in neuroblastoma and rhabdomyosarcoma cells, similar to tunicamycin or PNGase F treatment (Sieczkowski et al. 2010; Werner et al. 2013). The ABCB1 transporter exists in a full glycosylated 170-kDa form which upon deglycosylation shifts to the core glycosylated 140-kDa species (Loo and Clarke 1999). Although impaired glycosylation leads to a reduction in cell surface expression, the protein is still functional as this has been shown in various cellular systems (Germann et al. 1990; Kuchler and Thorner 1992; Loo and Clarke 1999).

The detection of ABCB1 by Western blot results in diffuse and fuzzy bands surrounding the 170- and 140-kDa region, due to the different levels of glycosylation (Loo and Clarke 1999; Greer and Ivey 2007; Seres et al. 2011). We also observed diffuse bands in particular in the presence of dolichol, which supports the conjecture that the level of glycosylation of the transporter is responsible for the overlap of the full and core glycosylated species (Fig. 4). In the presence of simvastatin, the overall amount of ABCB1 is reduced and thereby also the intensities of the 140-kDa core glycosylated band, which may explain that, in some cases, the shift from the 170 to the 140-kDa form is hardly pronounced. Another explanation for the heterogeneity of ABCB1 glycosylation is depending on cellular systems, in particular transformed versus untransformed cells (Ichikawa et al. 1991; Fakla et al. 1998). For example, the different glycosylation branches have been found to be alternatively bisected at ß(1-4)-N-acetyl-D-glucosamine residues (GlcNAc) in transformed cells. Nevertheless, we can exclude a methodical issue since other proteins are readily detected in these samples, like ERK1/2, cleaved PARP, or α-tubulin (Fig. 4).

The cell surface expression of ABCB1 in SH-SY5Y neuroblastoma cells is significantly reduced for longer incubation times in the presence of simvastatin (Fig. 1). This decline in ABCB1 expression is supported by Western blots of whole cell lysates probed for total ABCB1, which showed only a slight reduction after 24-h simvastatin exposure compared to 120-h incubations (Fig. 4a–d). This reduction of ABCB1 is considered to be not due to enhanced protein degradation, since the expression of the fusion protein YFP-ABCB1 is not further reduced in the presence of cycloheximide plus simvastatin compared to cycloheximide alone. Conversely, in the absence of transcriptional inhibition, simvastatin reduced YFP-ABCB1 within 48 h to the level obtained with cycloheximide alone. Moreover, this assumption is further corroborated by qPCR and a significant downregulation of mRNA for ABCB1, ABCC1, and ABCG2 over time (Fig. 1e, f). A compensatory upregulation of ABCC1 or ABCG2 is not observed, although these transporters share overlapping drug specificity and importance in multidrug resistance (Sharom 2008). In contrast, the mRNA for ABCC6 is hardly affected by simvastatin, indicating the specificity of the drug effect. A significant reduction of mRNA and functional activity for ABCB1 has been previously described in human hepatoma and blood cells (Rodrigues et al. 2006). Interestingly, in these cellular systems, 10 to 20 μM of atorvastatin caused an increase of ABCB1 on protein level.

Importantly, our findings have been verified by various methods (heterologous expression of YFP-ABCB1, FACS, and Western blots) and different antibodies against ABCB1 (C219, MRK16) in order to control for unspecific reactivity. In particular, heterologous expression of YFP-ABCB1 enabled direct monitoring in simvastatin-treated HEK-293 cells and corroborated downregulation in a concentration-dependent manner (Fig. 1d), while the anthracycline doxorubicin, a known inductor of ABCB1, significantly augmented YFP-ABCB1.

Besides plasma membrane, ABCB1 has been allocated to the endoplasmic reticulum, various endosomes, Golgi, and lysosomes, but not mitochondria (for review see (Fu and Arias 2012)). This intracellular organelle pattern reflects the protein synthesis, maturation and posttranslational modification, the translocation to the target membrane, and the degradation, respectively. Posttranslational modification of ABC transporters by *N*-glycosylation has been investigated intensively and plays a critical role in protein folding, protein export, and maintenance of protein stability (Richert et al. 1988; Asano et al. 1993; Schinkel et al. 1993; Lee et al. 2003; Zhang et al. 2004; Urquhart et al. 2005). Indeed, simvastatin reduced endogenous dolichol levels (Fig. 3) and thereby the prerequisite for proper protein glycosylation in the ER (Behrens and Leloir 1970). Others have shown that inhibition of glycosylation augments ubiquitination and degradation of ABCB1 (Kramer et al. 1993; Seres et al. 2011). Importantly, altered glycosylation does not affect the transport activity of ABCB1 (Germann et al. 1990; Kuchler and Thorner 1992; Schinkel et al. 1993; Loo and Clarke 1999; Seres et al. 2011), whereas ER trafficking is impaired (Loo and Clarke 1998). In non-polarized cells, approximately 30 % of the ABCB1 is found in EAA1- and Rab5-positive endosomes which shuttle between the plasma membrane and thereby control the cell surface expression of ABCB1 (Kim et al. 1997). It is known that Rab proteins control vesicle transport by interaction with microtubules and the actin cytoskeleton. Statins reduce isoprenylation of small G proteins, in particular, those interacting with the cytoskeleton (Kato et al. 2004; Gazzerro et al. 2012). This has been shown for Rab5 and Rab7 in lovastatin-treated FRTL-5 thyroid cells, which then accumulate in the cytosol (Laezza et al. 1998). This translocation from membrane fractions to the cytosol is explained by a decrease of geranylgeranylation and farnesylation, which serve as lipid anchor for membrane insertion. Coapplication of mevalonate, the product of the statin-inhibited HMG-CoA reductase, was sufficient to prevent cytosolic accumulation of Rab proteins (Laezza et al. 1998).

A link between reduced glycosylation and apoptosis is provided by the induction of BiP and CHOP, two sensitive markers for ER stress (Table 2). Thus, in a scenario of dolichol depletion, ER stress and reduced functional vesicular transport would explain pro-apoptotic events and less cell surface expression of ABCB1. However, reserve granules and intact transporters might be present in these endosomes which may serve as a compensatory reservoir within the first 24 h after simvastatin application. Consequently, in add-back assays, dolichol not only overcomes deglycosylation and downregulation of ABCB1 but also reduces simvastatin-induced apoptosis (Fig. 4).

Simvastatin-induced apoptosis is also shown in our in vivo findings. Two xenograft models confirm anti-tumor activity of simvastatin as well as ABCB1 downregulation. CD-1 Nu/Nu mice inoculated with rhabdomyosarcoma or neuroblastoma cells received clinically relevant simvastatin concentrations and showed a remarkable induction of apoptosis in both tumor tissues indicated by PARP and caspase 3 cleavages (Figs. 7 and 8). Most importantly, ABCB1 downregulation is found in the liver and tumor tissues but did not reach significance in neuroblastoma. Importantly, simvastatin is capable to trigger apoptosis in neuroblastoma and rhabdomyosarcoma. Moreover, the extent of apoptosis is comparable to cyclophosphamide and further amplified by the combination of the two drugs, although cyclophosphamide is not a substrate for ABCB1 (Fig. 8). This latter issue highlights the potential of simvastatin to act as an anti-tumor drug.

Statins are well established and safely used compounds in the treatment of hypercholesterolemia (Gazzerro et al. 2012). However, anti-tumor activities caught most attention in clinical trials and meta-analyses (Demierre et al. 2005; Gazzerro et al. 2012; Nielsen et al. 2012). Although available data are still controversial and conflicting, recent studies showed that statin use before a cancer diagnosis might be associated with lower risk of cancer incidence and cancer-related mortality (Gazzerro et al. 2012; Nielsen et al. 2012). Notably, all-cause mortality by statin users with cancer was reduced by 15 % (Nielsen et al. 2012).

Statins are also known to inhibit directly ABCB1, and for the various statins, the IC_50_ values match congruently the inhibition of ATP hydrolysis (Bogman et al. 2001; Wang et al. 2001; Goard et al. 2010; Sieczkowski et al. 2010; Werner et al. 2013). Consequently, statins enhance the intracellular accumulation of ABCB1 substrates like doxorubicin, which augments cytotoxicity in neuroblastoma and rhabdomyosarcoma cells and may provide the classical argument for usage in an anti-cancer therapy (Tamplin et al. 1954; Werner et al. 2004, 2013; Demierre et al. 2005; Goard et al. 2010; Sieczkowski et al. 2010; Tanaka et al. 2010).

In summary, simvastatin has a profound anti-tumor activity per se, which is now further highlighted by the fact that ABCB1 is downregulated in vitro and in vivo. Although simvastatin is well tolerated, these data also implicate that drug–drug interactions not only may take place on the level of CYP3A4 but also have the potential to interfere with drug extrusion via ABCB1. In fact, there is a strong overlap of compounds, which are substrates for both proteins (Sharom 2008; International Transporter C et al. 2010). Nevertheless, this study shows that it is feasible to downregulate ABCB1 in vitro and in vivo by simvastatin, which may act as a lead compound in novel anti-cancer therapies.

## Abbreviations

ABC transporter: ATP-binding cassette transporter
ABCB1: ABC transporter B1, P-glycoprotein
AFC: 7-Amino-4-trifluoro-methylcoumarin
ANOVA: Analysis of variance
CHOP: CCAAT/enhancer-binding protein (C/EBP)-homologous protein
DMEM: Dulbecco’s modified Eagle’s medium
ECL: Enhanced chemiluminescence
ER: Endoplasmic reticulum
FACS: Fluorescence-activated cell sorting
FPP: Farnesyl pyrophosphate
GGPP: Geranylgeranyl pyrophosphate
HEK cells: Human embryonic kidney cells
HMG-CoA reductase: 3-Hydroxy-3-methylglutaryl coenzyme A reductase
HPLC: High-performance liquid chromatography
HRP: Horseradish peroxidase
MDR: Multidrug resistance
PI: Propidium iodide
RIPA buffer: Radioimmunoprecipitation assay buffer
TLC: Thin-layer chromatography
